# Deep learning approaches for the detection of scar presence from cine cardiac magnetic resonance adding derived parametric images

**DOI:** 10.1007/s11517-024-03175-z

**Published:** 2024-08-06

**Authors:** Francesca Righetti, Giulia Rubiu, Marco Penso, Sara Moccia, Maria L. Carerj, Mauro Pepi, Gianluca Pontone, Enrico G. Caiani

**Affiliations:** 1 Department of Electronics, Information and Biomedical Engineering, Politecnico di Milano, P.zza L. da Vinci 32, 20133 Milan, Italy; 2 Centro Cardiologico Monzino IRCCS, Milan, Italy; 3 Istituto Auxologico Italiano IRCCS, San Luca Hospital, Milan, Italy; 4 Department of Innovative Technologies in Medicine and Dentistry, Università degli Studi “G. d’Annunzio” Chieti, Pescara, Italy; 5 Department of Biomedical Sciences and Morphological and Functional Imaging, “G. Martino” University Hospital Messina, Messina, Italy; 6 Department of Biomedical, Surgical and Dental Sciences, University of Milan, Milan, Italy

**Keywords:** Convolutional neural networks, Magnetic resonance image classification, Cardiac magnetic resonance imaging, Deep learning, Parametric images

## Abstract

This work proposes a convolutional neural network (CNN) that utilizes different combinations of parametric images computed from cine cardiac magnetic resonance (CMR) images, to classify each slice for possible myocardial scar tissue presence. The CNN performance comparison in respect to expert interpretation of CMR with late gadolinium enhancement (LGE) images, used as ground truth (GT), was conducted on 206 patients (158 scar, 48 control) from Centro Cardiologico Monzino (Milan, Italy) at both slice- and patient-levels. Left ventricle dynamic features were extracted in non-enhanced cine images using parametric images based on both Fourier and monogenic signal analyses. The CNN, fed with cine images and Fourier-based parametric images, achieved an area under the ROC curve of 0.86 (accuracy 0.79, F1 0.81, sensitivity 0.9, specificity 0.65, and negative (NPV) and positive (PPV) predictive values 0.83 and 0.77, respectively), for individual slice classification. Remarkably, it exhibited 1.0 prediction accuracy (F1 0.98, sensitivity 1.0, specificity 0.9, NPV 1.0, and PPV 0.97) in patient classification as a control or pathologic. The proposed approach represents a first step towards scar detection in contrast-free CMR images. Patient-level results suggest its preliminary potential as a screening tool to guide decisions regarding LGE-CMR prescription, particularly in cases where indication is uncertain.

**Graphical abstract:**

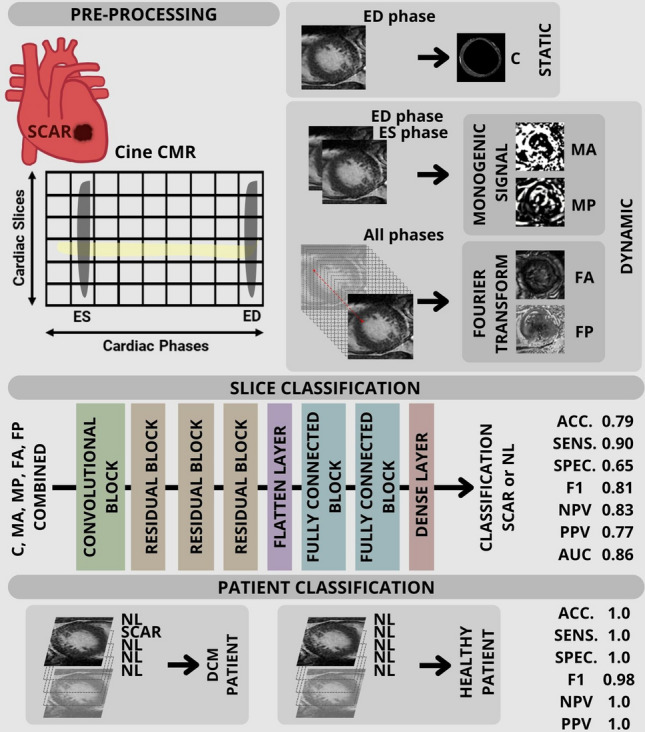

## Introduction

Ischemic cardiomyopathy (ICM) is the most common cause of heart failure. At its first stage, ICM causes a reversible loss in cardiac function due to reduced oxygenation. When ischemia is prolonged, irreversible damage to myocardial tissue occurs, leading to fibrosis through a tissue remodeling process. Myocardial fibrosis impacts the contractile properties of the affected area, setting the stage for increased arrhythmogenicity [1].

Cardiac magnetic resonance (CMR) with late gadolinium (Gd) enhancement (CMR-LGE) is the elective imaging modality for myocardial scar characterization [2–5]. CMR-LGE imaging relies on the different accumulation of Gd in different tissues, with a larger uptake in chronically damaged myocardium. This contrast-enhanced imaging modality relies on visual interpretation, being dependent on observer’s experience and expertise. Despite subjective factors that may lead to user-related measurement errors and high inter-observer variability, in clinical practice, the visual inspection of CMR-LGE represents the conventional technique to assess cardiac viability and to identify myocardial scars [6–8].

Recent studies have shown that CMR-LGE is highly contraindicated in patients with severe kidney disorders, and the exposure to Gd contrast agent has been associated with nephrogenic systemic fibrosis [9]. Studies also showed that Gd may represent a long-term risk factor as it can be retained in the cerebral and cerebellar nuclei by crossing the blood–brain barrier [10], and it can accumulate in the bone, skin, liver, and lungs [11]. For these reasons, Gd-based contrast agent administration should be considered carefully with respect to potential risks and benefits, and only used when required, as well as standard dosing should be used, and repeated administrations should be avoided unless necessary [12, 13].

Non-contrast CMR can be considered as an alternative [14], as the use of Gd-free steady-state free precession (SSFP) cine CMR pulse sequences could be explored to potentially identify scars in an indirect way. In fact, as the left ventricular (LV) wall motion and contractile properties are modified by the presence of nonviable scar tissue, researchers are investigating the potential of deep learning (DL) as a strategy to support the physician in identifying fibrotic tissue in the LV myocardial wall in Gd-free CMR cine sequences [15], thus overcoming the problem of Gd toxicity concerns [16].

Although previous methods [8, 16–20], based on capturing dynamic changes in videointensity across various spatial locations within the heart and temporal phases of the cardiac cycle, potentially enable the detection of myocardial fibrosis, they are still subject to limitations. In fact, due to the poorly contrasted scar tissue in cine frames, to apply this spatio-temporal learning paradigm, an increase in feature size is introduced, as they are extracted from all frames in the cardiac cycle, thus posing issues relevant to overfitting. In addition, hence optical flow-based methods for motion analysis are valuable, they could potentially be sensitive to through-plane motion artifacts, image quality, and brightness variation along the cine sequence [21, 22].

To overcome these issues, we hypothesized that the use of parametric images, derived from SSFP cine CMR images, integrating spatial and temporal information on LV wall motion in a compact way [23, 24], could overcome the intrinsic complexity of modeling motion deformation of the LV myocardium by synthetizing it into a single image [24–26].

Accordingly, our aim was to propose a novel DL approach, utilizing a convolutional neural network (CNN) that exploits the information from a single static end-diastolic (ED) frame together with multiple parametric images derived from the cine CMR loop, computed by two different approaches (i.e., Fourier transform and monogenic signal), to obtain a classification for each image slice, indicating the presence or absence of LV scar tissue.

To evaluate the effectiveness of this methodology, by testing various combinations of parametric images, its performance will be compared against the use of LGE images and expert interpretation, considered as the ground truth (GT). Such approach could be used to attract attention of the medical observer interpreting the cine CMR images towards those slices where the scar presence has been detected, thus serving as a support to the decision-making process of prescribing LGE imaging, in particular in cases in which the clinical indication is uncertain [14].

## Materials and methods

### Study population and image acquisition

This retrospective study included a cohort of consecutive patients who were referred for LGE-CMR imaging at the IRCCS Centro Cardiologico Monzino (Milan, Italy) between 2010 and 2016. Patients were excluded if standard contraindications to CMR-LGE existed, such as a glomerular filtration rate of ≤ 30 mL/min/1.73 m^2^. Institution’s ethical committee approved the protocol (ref. R659/17-CCM 698), and all patients gave written consent.

Images from 158 patients with ischemic dilated cardiomyopathy (DCM) showing the presence of fibrotic tissue in the LV myocardium, and from 48 control patients with a negative CMR-LGE, were studied. The main clinical and anthropometric parameters relevant to the enrolled subjects as a whole, and separately as DCM and control group, are reported in Table 1, together with the result of their statistical comparison (Mann–Whitney, or chi-square test).

**Table 1:** Clinical and anthropometric characteristics of patients: comparison of DCM and control groups using Mann–Whitney and Chi-Square Tests (P-Values). Continuous variables are presented as medians with first and third quartiles (Q1-Q3). Categorical variables are presented as percentages (%)

Characteristics	All (206)	DCM (158)	CTRL (48)	*P* value
Age, years (Q1–Q3)	67.0 (56.0**–**72.8)	68.0 (59.0–73.8)	54.5 (34.8–70.0)	< 0.0001
Sex, %				
female	20.4	15.2	37.5	0.002
male	79.6	84.8	62.5	
Weight, Kg (Q1–Q3)	77.0 (69.0**–**86.0)	79.0 (70.0**–**87.0)	70.0 (60.5**–**83.5)	0.013
Height, cm (Q1–Q3)	171.5 (165.0**–**178.0)	172.0 (165.0**–**178.0)	170.0 (165.0**–**180.0)	0.671
BMI, Kg/m^2^ (Q1–Q3)	26.0 (23.3**–**28.9)	26.1 (24.3**–**29.3)	24.0 (21.8**–**27.0)	0.001
BSA, m^2^ (Q1–Q3)	1.9 (1.8**–**2.0)	1.9 (1.8**–**2.0)	1.8 (1.7**–**2.0)	0.238
Familiar history, %	25.5	27.7	15.2	0.199
Smoking, %	33.0	37.4	12.1	0.009
Hypertension, %	59.0	65.8	27.3	< 0.0001
Hyperlipemia, %	52.7	57.4	30.3	0.008
Diabetes, %	29.8	33.5	12.1	0.026
Beta-blockade, %	77.1	88.4	24.2	< 0.0001
ACE inhibitor/AT1 blockade, %	67.6	76.8	24.2	< 0.0001
Diuretics, %	63.8	76.8	3.0	< 0.0001
Ca blockade, %	8.0	8.4	6.1	0.920
Anti-thrombotic agents, %	77.1	89.0	21.2	< 0.0001
Nitrates, %	19.3	23.2	0.0	0.005
Statin, %	63.3	69.0	36.4	0.001
Antiarrhythmic, %	29.8	31.6	21.2	0.330
LV EDVi, mL/m^2^ (Q1–Q3)	104.0 (85.6**–**130.3)	114.0 (94.9**–**144.9)	76.3 (63.8**–**88.4)	< 0.0001
LV ESVi, mL/m^2^ (Q1–Q3)	72.1 (45.1**–**95.1)	78.7 (62.8**–**105.5)	32.1 (26.5**–**38.1)	< 0.0001
CMR LV EF, % (Q1–Q3)	32.6 (25.1**–**44.9)	30.4 (23.2**–**35.2)	57.0 (55.0**–**63.5)	< 0.0001
CMR LV SV, mL (Q1–Q3)	36.8 (29.4**–**46.1)	34.5 (27.3**–**41.4)	48.7 (41.0**–**54.9)	< 0.0001
LV mass ind, g/m^2^ (Q1–Q3)	65.5 (52.1**–**81.0)	70.0 (58.8**–**85.9)	50.4 (44.1**–**59.0)	< 0.0001
RV EDVi, mL/m^2^ (Q1–Q3)	64.5 (52.9**–**81.0)	60.9 (50.9**–**76.9)	76.1 (66.5**–**85.9)	< 0.0001
RV ESVi, mL/m^2^ (Q1–Q3)	29.6 (22.7**–**40.4)	28.4 (22.1**–**40.5)	35.4 (24.8**–**39.8)	0.130
CMR RV EF, % (Q1–Q3)	53.2 (44.5**–**61.6)	50.9 (41.0**–**61.1)	59.0 (53.0**–**63.0)	0.0004
CMR RV SV, mL (Q1–Q3)	53.2 (43.6**–**70.8)	56.0 (44.0**–**73.3)	46.3 (40.7**–**54.8)	0.003
LGE ischemic mass, g (Q1–Q3)	25.5 (15.5**–**37.9)	25.5 (15.5**–**37.9)	-	-

All CMR acquisitions were performed using a 1.5 T scanner (Discovery MR 450, GE Healthcare, Milwaukee, Wisconsin, USA), using phased-array surface receiver coils, and electrocardiogram triggering. Breath-hold SSFP cine imaging was performed in vertical and horizontal long-axis orientations as well as in short-axis, using the following parameters: field of view (FOV) 380 × 380 mm^2^, repetition time 3.2 ms, echo time 1.4 ms, flip angle 50°, image matrix size 224 × 256 pixels, bandwidth 488.3 Hz/pixel, and slice thickness 8 mm with no gap.

In addition, a contrast-enhanced, breath-hold, segmented T1-weighted inversion-recovery gradient-echo sequence (FOV 380 × 380 mm^2^, repetition time 6.6 ms, echo time 1.5 ms, flip angle 20°, image matrix size 224 × 192 pixels, bandwidth 122.1 Hz/pixel, slice thickness 8 mm) was used: LGE imaging was performed 10 to 20 min after the administration of an intravenous bolus of 0.1 mmol/kg of Gd-based contrast agent (Gadovist; Bayer AG, Berlin, Germany) at a flow rate of 3 ml/s, followed by 20 ml of saline flush with the same rate. Inversion time was individually adapted to null the signal of remote myocardium (220 to 300 ms) [27].

### Ground truth assessment

All images were analyzed using cvi42 cardiac software (version 5.11, Circle Cardiovascular Imaging Inc., Calgary, Canada) by an expert cardiologist (EACVI Level III CMR certified). For each patient, short-axis cine and LGE were extracted at matched anatomical slices.

On the stack of LGE images, the LV endocardium and epicardium borders were traced, and the myocardial fibrosis was outlined by the expert cardiologist through manual contouring, after appropriate setting of the display window level and width. Subsequently, each LGE image was automatically labeled based on the presence or absence of the scar in it, and this label was used as GT.

Additionally, in the matched cine short-axis view, the LV endocardium and epicardium borders at ED frame, automatically selected as the one with the largest LV blood pool at the mid-ventricular level, were manually contoured by the expert to include papillary muscles and trabeculations as part of the LV cavity, in agreement with recent guidelines [28–30].

### Deep learning model

#### Data pre-processing and augmentation

To reduce the processing area, CNN computational cost and memory requirement, in accordance with literature, a squared bounding-box including the whole LV myocardium was automatically retrieved and used to crop each ED image. To guarantee better generalization to unseen data, reducing overfitting, and preventing gradient-related issues, images were normalized to achieve zero mean and unit standard deviation, and resized to 224 × 224.

The dataset consisted of a total number of 1793 ED cropped images, of which 1047 with scar (SCAR) from the 158 DCM patients, and 746 without (NL) from the 48 control patients, based on the GT interpretation.

**Fig. 1:**
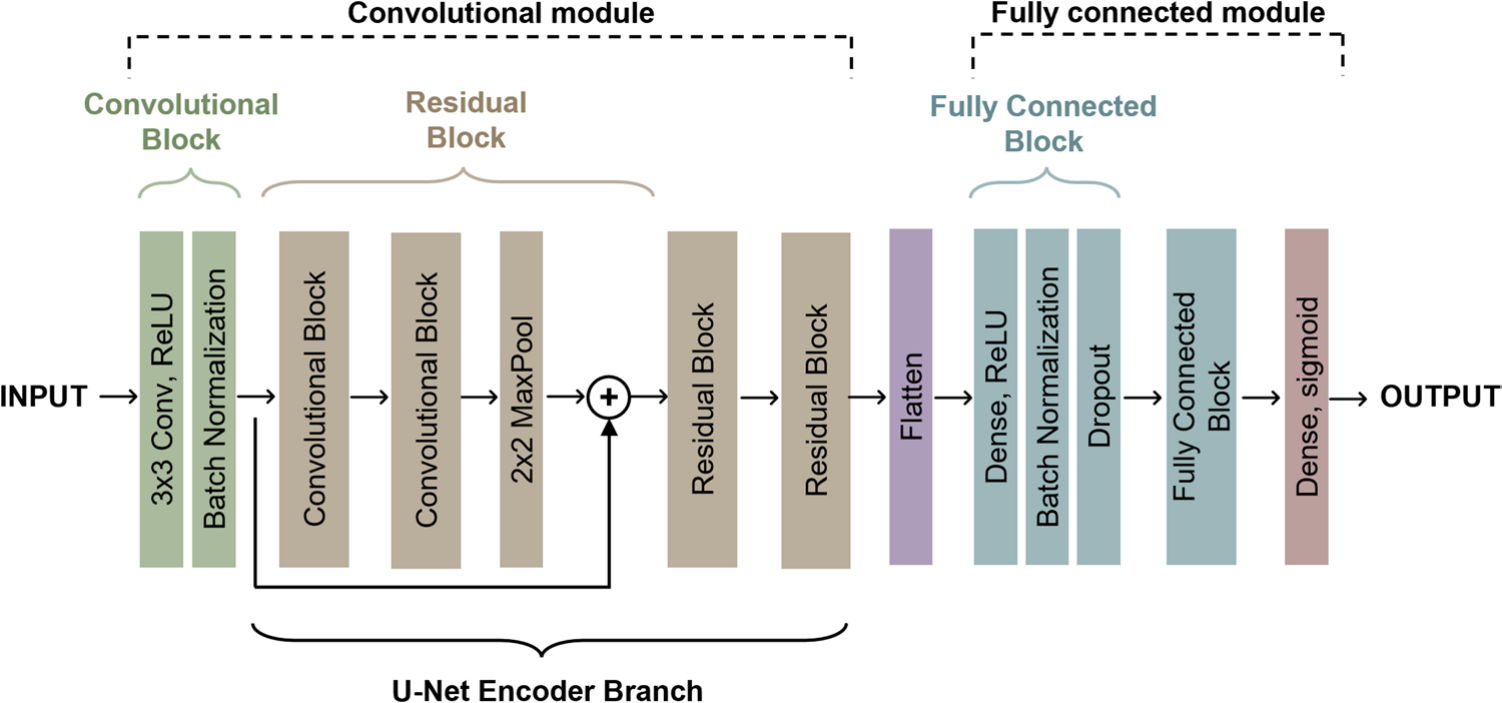
Sequence of basic components constituting the proposed CNN: initial convolutional block (composed of a convolutional layer and a batch normalization layer), three residual blocks (each one composed of two convolutional blocks followed by a max pooling layer with the addition of a skip connection), a flatten layer, two fully connected blocks (each composed of a dense layer, a batch normalization layer and a dropout layer), and a final dense layer

Our CNN embedded the encoding branch of U-Net [31], with one convolutional block followed by three residual blocks characterized by skip connections that connect the input of the block directly to the next one. Figure 1 shows the architecture of the proposed CNN.

The first initial convolutional block was made of a convolutional layer (number of kernels = 64, kernel size 3 × 3) with batch normalization [32]. The three residual blocks differed by filter size, respectively 128, 256, and 512, and had the following structure: two convolutional blocks (as in the initial convolutional block) and a max-pooling layer (size 2 × 2) [33]. A skip-connection was introduced between the input and the output of the residual block. All convolutional layers were characterized by a kernel size 3 × 3, rectified linear unit (ReLU) [33] as activation function and He uniform as kernel initializer. This convolutional module was connected through a tensor flattening layer to two sequential fully connected (FC) layers with 256 and 128 neurons, respectively, each followed by a batch normalization layer and a dropout layer (rate 0.2) [33, 34]. A final dense layer with one node and sigmoid activation function was used for binary classification.

#### Parametric images computation

The parametric imaging technique is based on the measurement of signal variability within the same pixel coordinates over the cardiac cycle to capture dynamic information of the LV myocardium [25]. For each extracted signal over time, amplitude and phase were computed as representative of the myocardium wall motion throughout the cardiac cycle, according to the applied approach (i.e., Fourier or monogenic signal analysis).

##### Fourier analysis

Along each cine loop, for each image pixel coordinate, the time series of video intensity values was obtained [24, 35] and approximated to a best-fit curve by using the standard least squares analysis. As during the cardiac cycle the signal is assumed to be periodic, based on the Fourier theorem, this continuous and periodic function can be decomposed into a linear combination of harmonics and represented by the Fourier series (Eq. 1).

1 $$f\left(t\right)= {A}_{0}+ \left({A}_{1} \times \text{sin}\left(\omega t+ {P}_{1}\right)\right)$$

**Fig. 2:**
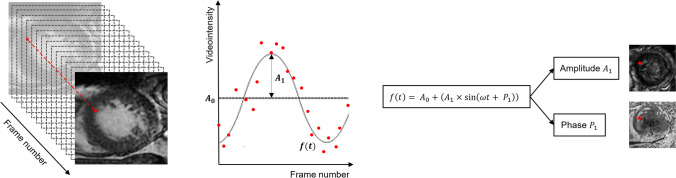
The time series of videointensity values is approximated to a periodic function (oscillations of amplitude ${A}_{1}$ around the mean value ${A}_{0}$) that can be represented with the Fourier series. Amplitude and phase for each pixel are obtained from the first harmonic

For each pixel at a particular location, focusing on the fundamental series of order *n* = 1, the amplitude ${A}_{1}$ and the phase ${P}_{1}$ of this first harmonic were exploited to create the two corresponding parametric images. This process was applied for each cine-loop available from the study (Fig. 2).

##### Monogenic signal analysis

**Fig. 3:**
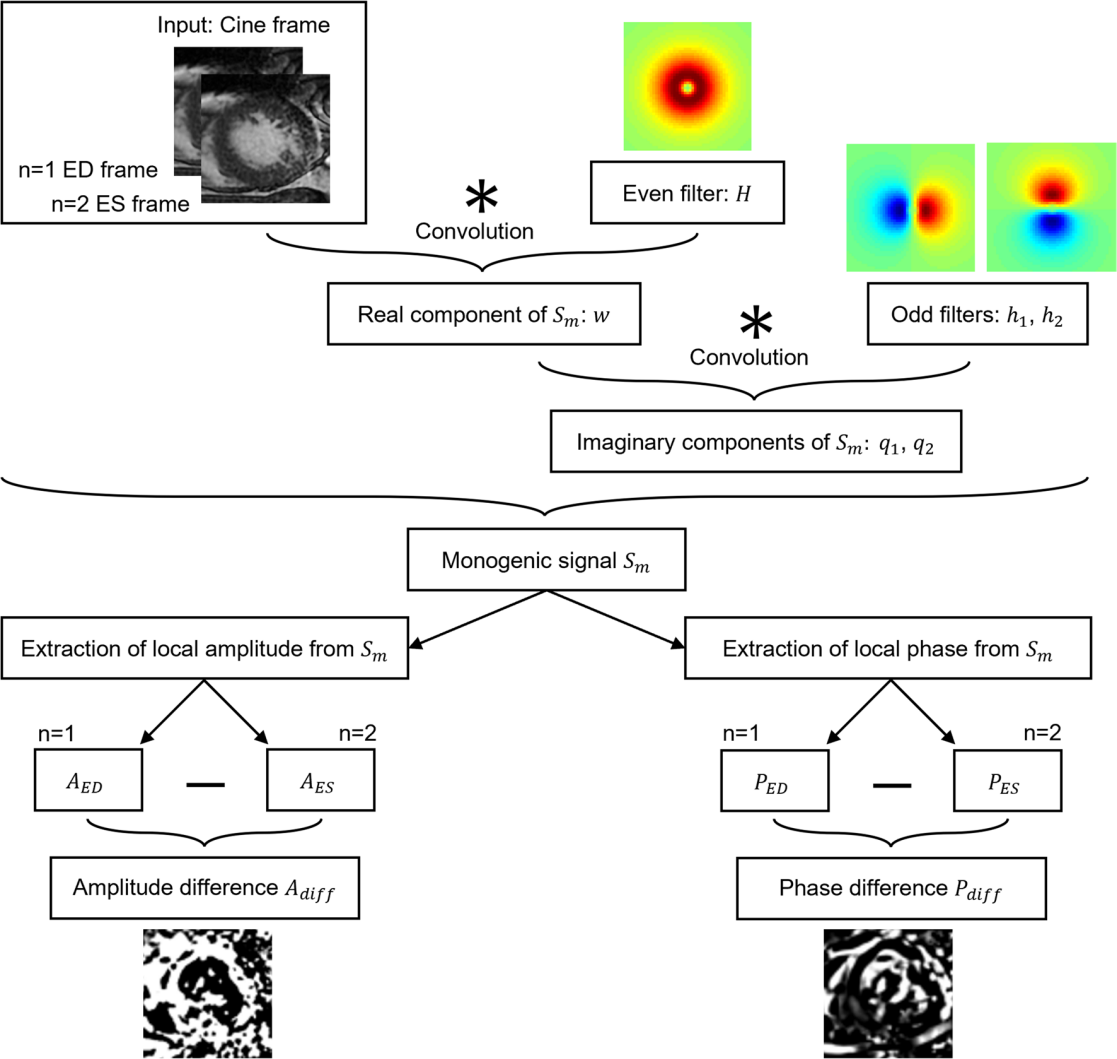
Workflow to obtain the monogenic signal ${S}_{m}$ and derive parametric amplitude and phase images from it (see text for details)

In the second approach, based on the monogenic signal, information of the myocardial displacement was obtained considering only the ED and the end-systolic (ES) frames for each cardiac cycle, following the method described in [21, 36]. In brief, the monogenic signal ${S}_{m}$ (Eq. 4) was obtained by first convolving the image $I$ with the even log-Gabor filter $H$ (Eq. 2), and then convolving its result $w$ with two odd filters ${h}_{1}$ and ${h}_{2}$ (Eq. 3), calculated applying to $H$ the Riesz transform (Fig. 3).

2 $$w=I\ast H$$

3 $$\begin{array}{c}{q}_{1}=w\ast {h}_{1}\\ {q}_{2}=w\ast {h}_{2}\end{array}$$

4 $${S}_{m}=w+i\ast {q}_{1}+j\ast {q}_{2}$$

The signal ${S}_{m}$ was then decomposed in a standard spherical polar coordinates system to obtain its amplitude (Eq. 5) and phase (Eq. 6) values.

5 $$A= \sqrt{{w}^{2}+ {q}_{1}^{2}+ {q}_{2}^{2}}$$

6 $$P=\text{arctan}\left(\frac{\sqrt{{q}_{1}^{2}+ {q}_{2}^{2}}}{w}\right)$$

The corresponding parametric images of amplitude (Eq. 7) and phase (Eq. 8) were obtained computing the difference between the corresponding ED and ES values:

7 $${A}_{\text{diff}}= {A}_{ED}- {A}_{ES}$$

8 $${P}_{\text{diff}}= {P}_{ED}- {P}_{ES}$$

### Model inputs

Five different types of parametric images were considered for each patient for each slice: the region of interest in correspondence to the myocardium of the ED cine frame (C), the amplitude (FA) and phase (FP) from Fourier analysis, the amplitude (MA), and phase (MP) from monogenic signal. As regards the ED cine frame, the LV endocardium and epicardium contours traced by the expert were used as a mask to maintain the videointensity content only in the region of interest in correspondence to the myocardium, and zero values elsewhere.

**Fig. 4:**
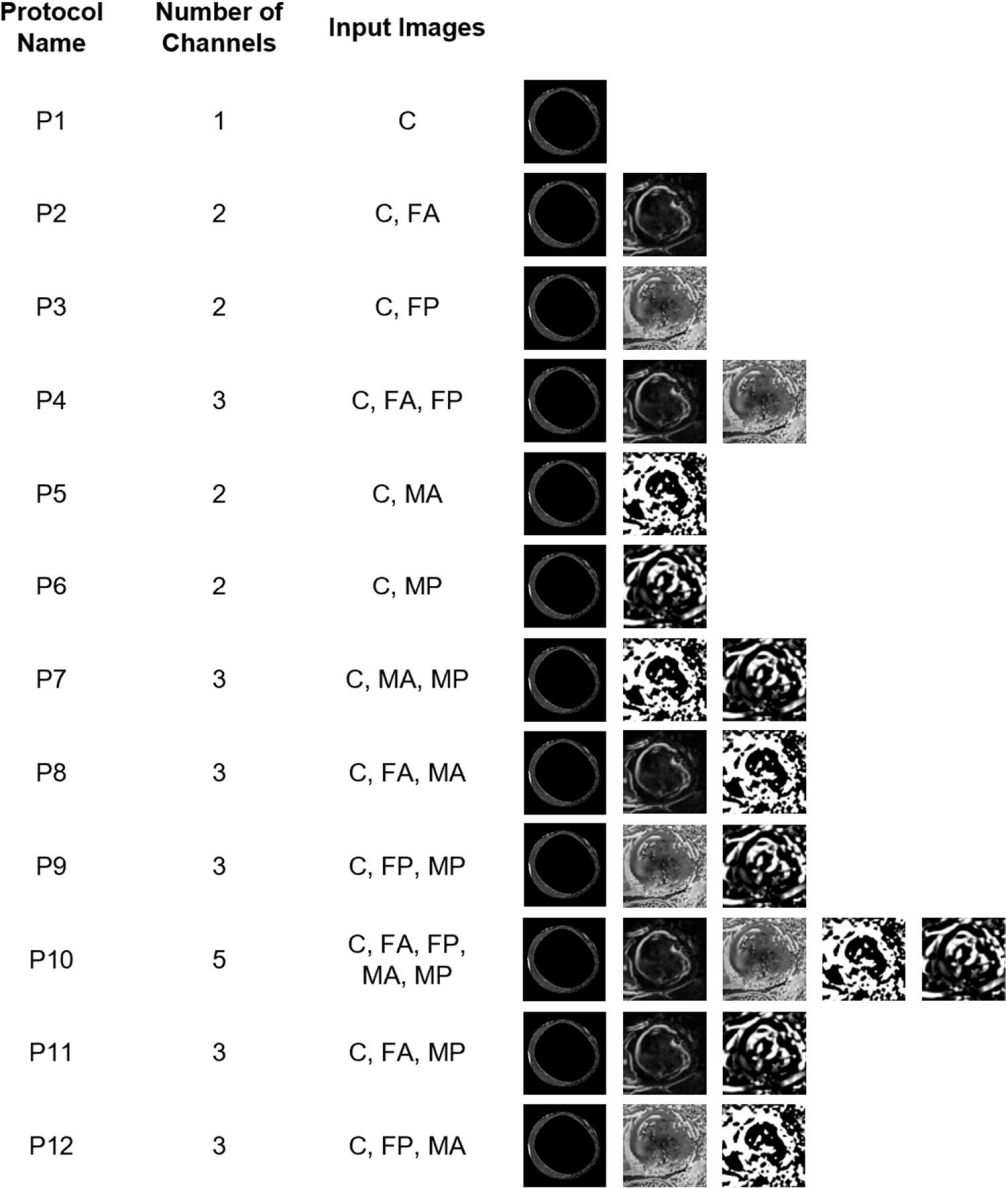
The evaluation protocols are summarized in the figure showing the protocol name, the number of channels, and the combination of input images: original cine images (C), amplitude of parametric based on Fourier analysis (FA), phase of parametric based on Fourier analysis (FP), amplitude of parametric based on monogenic signal (MA), and phase of parametric based on monogenic signal (MP)

Different combinations of those images were composed to create n-channel input with whom to train and validate the CNN by computing the relevant performance compared to the GT label, thus generating different evaluation protocols, as shown in Fig. 4. In the first one (P1), only the single ED frame (static information) was included as reference: in this way, the added value of including motion features from consecutive cine frames obtained through parametric images (dynamic information) by Fourier (P2, P3, P4) or monogenic signal (P5, P6, P7) analysis could be derived. In P8 and P9, only amplitude and phase images, respectively, were added, while in P10, all parametric images were jointly utilized. Additionally, in P11 and P12, a combination of FA with MP, and of FP with MA, respectively, were considered.

### Model evaluation and experimental protocol

The dataset was randomly divided (patient-wise) into training and test set by stratifying the two groups according to the global label of the patient (SCAR and NL) and allocating 80% of the initial patients to the training set (164 patients, resulting in 840 SCAR and 587 NL images) and 20% to the test set (42 patients, resulting in 207 SCAR and 159 NL images).

The training set was further divided (patient-wise) to allocate 15% of these patients for validation (25 patients, resulting in 132 SCAR and 83 NL images) and 85% for the actual training set (139 patients, resulting in 708 SCAR and 504 NL images). The training dataset was augmented applying random image flipping, width and height shifting (randomly from 0 to 0.2), and rotation (randomly from − 20 to 20°).

The performance of each protocol was evaluated by considering the target label of each individual image for the classification of SCAR and NL images. For CNN training, stochastic gradient descent was used as the optimizer (with 0.001 as learning rate) to minimize the binary cross-entropy. The maximum number of epochs was set to 200, with an early stopping if a plateau of validation loss was reached over 16 consecutive epochs. The batch size was fixed to 32 for the training set.

To evaluate the performance of the model on the test set, the confusion matrix was computed and accuracy (acc), F1, sensitivity and specificity, positive (PPV) and negative (NPV) predictive values, together with the area under the curve (AUCs) of the receiver operating characteristic (ROC) curve, were derived.

In the second step, the performance of the CNNs obtained in the defined protocols was tested at patient-level by considering as target label the existence of at least one scar (i.e., a patient in the DCM group) or none (i.e., a patient in the control group) in the acquired slices. Patient-wise accuracy was calculated as the number of correctly classified patients divided by the total number of patients in the test set, and reported together with F1, sensitivity, specificity, PPV, and NPV.

## Results

**Fig. 5:**
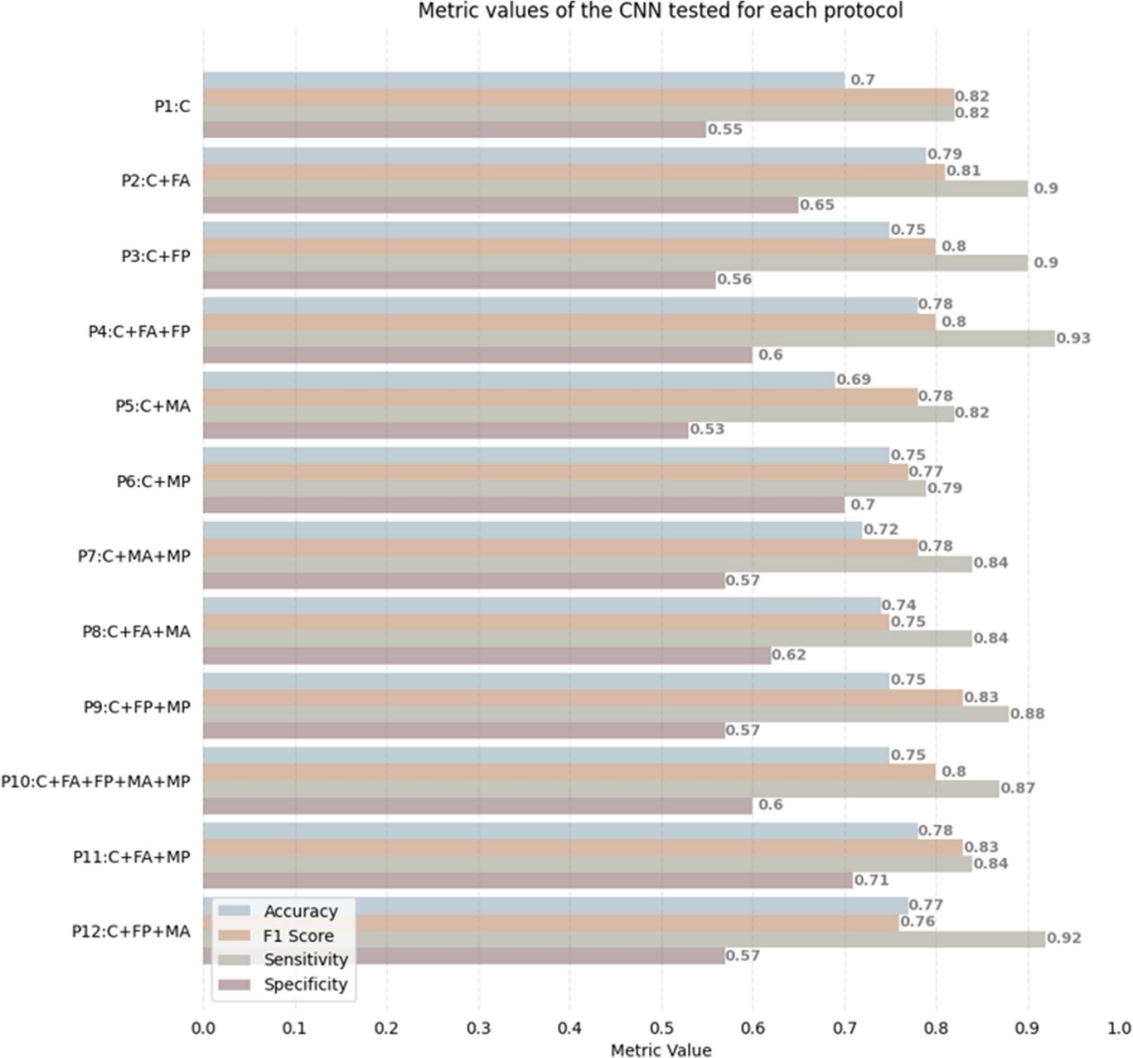
Results in terms of accuracy, F1 score, sensitivity and specificity obtained by testing the CNN with the protocol P1, containing static information, and with the protocols from P2 to P12, including the addition of dynamic information

The different performance, in terms of test set accuracy, F1, sensitivity and specificity of the CNN trained with the examined protocols are shown in Fig. 5, while relevant NPV and PPV are reported in Table 2. The accuracy (acc) obtained using only the static information (P1) was lower to all but one (P5, adding the amplitude of monogenic signal) the remaining protocols. P2, which adds the Fourier parametric amplitude, reached the highest values (acc 0.79). Adding also the Fourier phase information did not further improve the results (P4, acc 0.78), while for monogenic signal the only addition of the phase image resulted in a better performance (P6, acc 0.75) than combining both amplitude and phase (P7, acc 0.72). Considering only amplitudes FA and MA (P8, acc 0.74), or phase FP and MP (P9, acc 0.75) parametric images resulted in intermediate values. The combination of Fourier amplitude and monogenic phase (P11, acc 0.78), as well as of Fourier phase and monogenic amplitude (P12. acc 0.77) did not further improve the performance. Finally, the inclusion of all the parametric images (P10, acc 0.75) appeared constrained to the lower results obtained by the monogenic approach.

**Table 2:** Results in terms of PPV and NPV predictive values obtained by testing the CNN with the protocol P1, containing static information, and with the protocols from P2 to P12, including the addition of dynamic information

	P1	P2	P3	P4	P5	P6	P7	P8	P9	P10	P11	P12
NPV	0.70	0.83	0.81	0.86	0.69	0.72	0.73	0.74	0.79	0.79	0.77	0.84
PPV	0.71	0.77	0.73	0.75	0.70	0.78	0.71	0.74	0.73	0.74	0.79	0.74

**Fig. 6:**
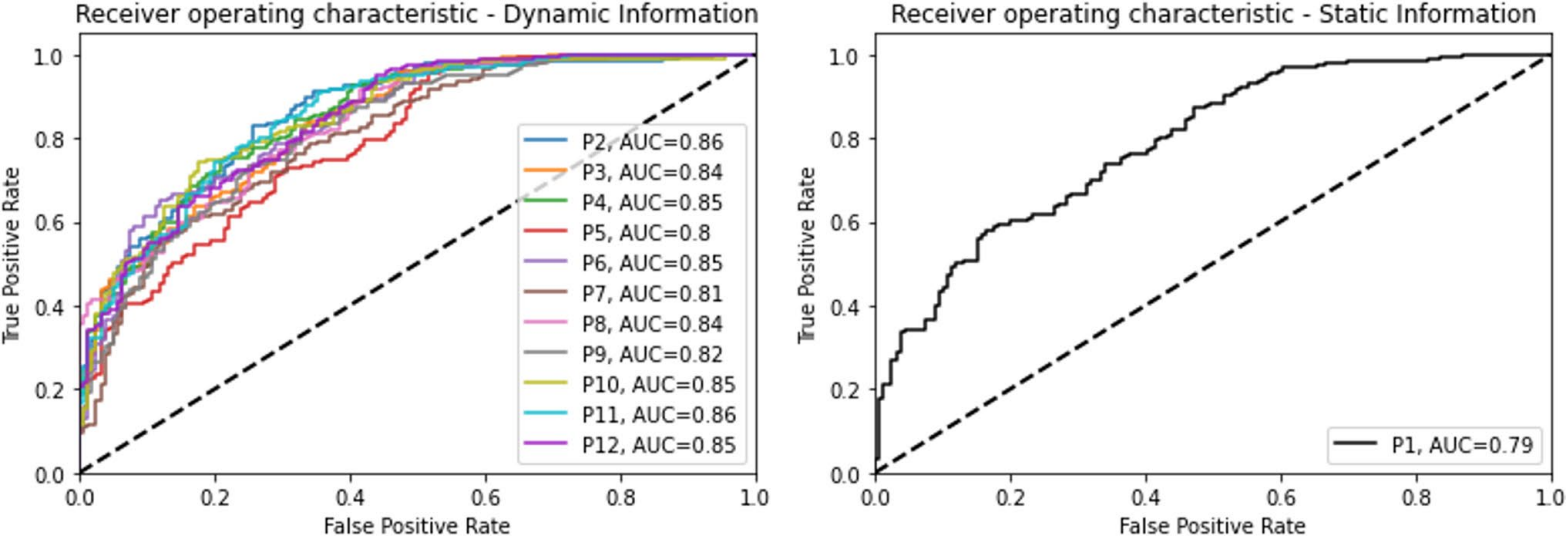
ROC curves with corresponding AUC of the CNN tested with the protocol P1 (static information) and with the protocols from P2 to P12 (a*ddition of dynamic information)*

The AUCs for all protocols, with similar findings, are shown in Fig. 6.

When comparing the performance obtained using the Fourier and the monogenic signal analyses, Fourier approach showed the best accuracy and AUCs results (P2, acc 0.79; AUC ROC, 0.86).

### Patient-level analysis

**Fig. 7:**
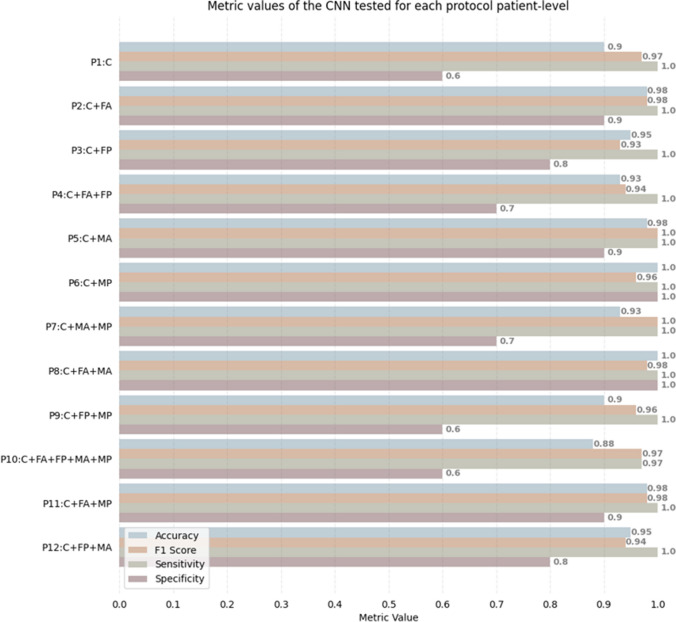
Results in terms of accuracy, F1 score, sensitivity, and specificity obtained by testing the CNN at patient-level

The accuracy, F1, sensitivity and specificity values of the proposed CNN considering the global label of the patient are shown in Fig. 7, and relevant NPV and PPV are reported in Table 3. Results were very good for all the considered protocols, with the worst performance in P10 (all parametric images together), and the best in P6 and P8, in which all patients were correctly classified as DCM or control, thus achieving an accuracy equal to 1.0. When considering P2, P5, and P11, a quasi-excellent performance (acc 0.98) was found, with 41/42 patients classified correctly and only one false positive.

**Table 3:** Results in terms of PPV and NPV predictive values obtained by testing the CNN at patient-level

	P1	P2	P3	P4	P5	P6	P7	P8	P9	P10	P11	P12
NPV	1.0	1.0	1.0	1.0	1.0	1.0	1.0	1.0	1.0	0.86	1.0	1.0
PPV	0.89	0.97	0.94	0.91	0.97	1.0	0.91	1.0	0.89	0.89	0.97	0.94

## Discussion

In this study, a DL method was proposed for the classification of the presence or absence of scar in LV myocardial tissue from Gd-free cine CMR images using the static ED frame combined with a diverse set of parametric images, each defining distinct protocols.

For each slice, the inclusion of motion features computed from consecutive cine frames proved to be more effective with respect to considering the ED frame only. Specifically, the parametric images synthetize the abnormal movement of the LV myocardial wall in presence of fibrotic tissue [37] inside one single frame, thus allowing the network, taking advantage from the encoder branch of the U-Net, to extract additional features useful to better distinguish healthy myocardium from the scarred one, without the risk of drastically increasing the feature size [24–26].

We previously tested a similar approach using a Random Forest (RF) classifier [35] in a small group of 40 patients, in which a parametric image was computed using the Fourier analysis. In this work, we extended our study by considering a wider population, by applying a CNN, and by comparing the performance of using several combinations of parametric images, computed both with the Fourier analysis and the monogenic signal [21, 38].

Unlike the Fourier analysis, the monogenic signal computation is not based on the hypothesis of periodicity of the videointensity signal through the cardiac cycle, and also has the advantage of requiring less computational resources, being based on the convolution of the ED and ES frames with specific filters. Despite these benefits, the approach based on the Fourier analysis provided better results, probably due to the punctual evaluation pixel by pixel of the videointensity oscillations along the entire cine sequence, thus allowing the extraction of more detailed and comprehensive dynamic information.

Among the multitude of benefits provided by the integration of residual blocks into the CNN architecture, one of the most significant is their ability in addressing the vanishing gradient problem [39, 40]. In fact, in deep neural networks, the gradient of the loss function approaches zero during the backpropagation, making the early layers parameter update negligible. However, the inclusion of residual blocks overcomes this limitation providing alternative shortcut paths for the propagation of the gradient. In advance, CNN including residual blocks can learn intricate and complex mapping, capturing hierarchical and multi-scale features from the input data. In fact, by transmitting information directly to deeper layers, skip connections allow the network to simultaneously preserve fine-grained details while progressively extracting highly abstract features delving deeper into the network. This combination of information at various scales and levels of abstraction enables the network to tackle challenging image classification tasks [40]. Furthermore, skip connections enhance the generalization capabilities of deep neural networks, grasping meaningful representations of underlying patterns in data without becoming overly specific to the training dataset. In this way, the CNN with residual blocks becomes more robust and reliable in real-world applications thus preventing overfitting [39].

Given the network’s capability to classify slices as either SCAR or NL (with an accuracy of 0.79 and an AUC ROC of 0.86), the proposed method could serve as a tool for clinicians to focus their attention on slices representing myocardium with potential scar tissue, to confirm or not such classification by visualizing the corresponding dynamic cine images to check for wall motion abnormalities, and subsequently prescribe CMR-LGE imaging for possible confirmation of the diagnosis.

Similarly, by repeating this classification for all the slices of the cine CMR exam, it could be possible to preliminary classify a patient as a control or pathologic based on the presence or not of a scar in the whole myocardium. Specifically, using P6 and P8 combinations, no false negative and false positive classifications were present when considering such patient-level analysis. Due to its high sensitivity and specificity, the proposed DL-model could represent a possible preliminary screening tool to serve as support to the decision of performing or not LGE in those cases in which its clinical indication is uncertain [14]. Indeed, several studies showed that about 50% of patients with a specific type of cardiomyopathy have no scar but undergo repeated Gd-based CMRs throughout their life [8].

On the other hand, in patients in which both cine CMR and the CMR-LGE images have been acquired, the proposed approach could represent a valuable tool to facilitate and speed up the clinician in the examination of the Gd-enhanced images, by flagging those slices classified as having a possible scar for further confirmation.

In specific clinical applications, such as cardiac resynchronization therapy, where it is not necessary to quantify the scar area but to localize its presence along the slices, our approach could provide insightful information. As a scar in the basal wall (i.e., over the LV pacing site) has been shown to be associated to an adverse impact on long-term prognosis [8, 41, 42], a preliminary assessment for the presence of a scar in that slice could be initially performed without contrast medium using the proposed approach.

Table 4 shows the comparison of the results obtained in this work with similar studies using machine learning (ML) or DL methods for scar detection from contrast-free cine sequences.

**Table 4:** Comparison of the results obtained using ML and DL models in the current state of the art

Reference	Authors	No. of subjects	Model	AUC	Accuracy	Sensitivity		Specificity	Approach description
This work	Righetti F. et al. (2024)	206	CNN with embedded U-Net encoder branch	Slice-level					Automatic motion feature extraction with two parametric images of amplitude and phase using Fourier analysis and monogenic signal
				0.86	0.79	0.90		0.65	
				Patient-level					
				-	1	1		1	
[16]	Larroza A. et al. (2018)	50	SVM (support vector machine)	0.85	-	0.83		0.73	LBP extracted from cine sectors using 2D + *t* texture analysis
[17]	Zhang N. et al. (2019)	299	RNN	0.94	-	0.90		0.99	Global and local motion features extracted using optical flow
[18]	Xu C. et al. (2018)	165	LSTM-RNN	-	0.95	0.91		0.99	Image-based global motion features (optical flow) and patch-based local motion features (intensity changes)
[19]	Xu C. et al. (2020)	280	PSCGA	0.90	0.97	0.91		0.99	LGE-equivalent image generation and simultaneous scar segmentation. Spatial and temporal features automatic extraction through SCLN (sequential causal learning network)
[20]	Zhang Q et al. (2022)	843	VNE (virtual native enhancement) with GAN	-	0.84	0.77		1	Scar detection in LGE-like image generated combining cine images with T1 maps
[8]	Fahmy A. S. et al. (2022)	993	LR	0.81	0.65		0.91	0.42	DL features automatically extracted by a pre-trained network and concatenated to radiomics features to create a combined set
[35]	Moccia S. et al. (2020)	40	RF (random forest)	0.75	-	0.70		0.69	Spatial and temporal features extracted using LBP and two parametric images of amplitude and phase using Fourier analysis

In particular, Larroza et al. [16] used a ML classifier to identify the presence of nonviable segments in LV sectors using local binary patterns (LBP), computed from the cine dynamic images using a 2D + *t* approach, as textural descriptors.

Applying a DL approach, Zhang et al. [17] proposed a recurrent neural network (RNN) capable of extracting global and local motion features using optical flow and achieving an AUC of 0.94 in the scar detection task. Xu et al. [18] introduced a long short-term memory (LSTM) RNN to demonstrate the correspondence between motion features extracted from non-enhanced images and tissue properties allowing to determine the tissue identity in each pixel from its motion pattern, further proposing a progressive sequential causal generative adversarial network (PSCGAN) for LGE-equivalent image generation and simultaneous scar segmentation, resulting in an AUC of 0.90 [19].

More recently, Zhang et al. [20] proposed a method to generate virtual LGE-like images combining cine images and T1-maps through a learning-based strategy, thus reaching an overall accuracy of 0.84 in detecting scars, but being limited to a single frame analysis and not exploiting the LV motion features. Fahmy et al. [8] performed scar detection in cine sequences using a logistic regression (LR) classifier exploiting a set of radiomics features combined with DL features obtained through a pre-trained network, reaching an AUC of 0.81, without considering the motion information.

The results obtained in this study at slice-level with CNN relying on parametric images were comparable to [16] obtained with Support Vector Machines (SVM). On the contrary, our performance at slice-level appears slightly inferior to studies in which more elaborate network architectures were used for automatic motion feature extraction [19, 20] or used in combination with local and global motion features extracted through optical flow [17, 18]. On the other hand, when compared to [8, 35] where the classification models were based on ML approach, this work showed higher performance. Furthermore, results of our network reached the highest accuracy, sensitivity and specificity when considering the analysis at patient-level.

### Limits and future developments

A possible limitation of our study concerns the relatively small size of the dataset. Collecting more data to expand the input dataset would improve the performance of the network. Moreover, the GT definition was constrained by the limited availability of specialized experts, due to its time-consuming aspect, thus relying solely on myocardial fibrosis outlined by one expert cardiologist through manual contouring on the LGE images; future studies should be designed to include multiple independent annotations from which to derive consensus, or considering interobserver variability in the definition of the GT, to widen the reliability and applicability of the findings. Another limit of this work is that only a single trial was performed for each protocol. As it is well known, there is a certain component of variability in neural networks training; therefore, to make the conclusions more robust, several trials could be run to compute the mean and standard deviation. Similarly, we opted for a simple validation method based on a random split instead of multifold cross-validation due to the associated computational complexity and the significant increase in processing time in this specific case, which included the implementation of multimodal protocols. Future research could incorporate multifold cross-validation to more rigorously validate the models. In addition, since images were acquired through one single scanner, it would be beneficial to consider the influence of different CMR acquisition systems, to test for external validity of the proposed CNN.

An additional limitation is that the proposed method does not provide scar segmentation and quantification of its area, but only the possible presence of the scar in the slice of interest (or at patient-level). However, this could be seen as a first step towards that goal that would require a larger and more extensive validation.

Further developments may involve the implementation and comparison of new techniques for including temporal information, such as optical flow or new types of parametric images.

## Conclusion

The possibility of using Gd-free cine CMR images to classify the presence of scar tissue at both slice and patient-level using a DL approach, utilizing a custom-made convolutional neural network (CNN) that exploits several parametric images to capture dynamic wall motion information, was tested.

The innovation in this study primarily lies in the different methods (i.e., Fourier analysis and monogenic signal) to generate the parametric images, and the comparison of their multiple combinations to define the most performant one compared to the expert interpretation of LGE images. Globally, the use of parametric images in the CNN improved the accuracy of properly classifying a slice compared to the use of only the static ED image, with the best performance obtained by adding the parametric image of the Fourier’s transform module. At patient-level, an accuracy of 1.0 in classifying normal or pathologic patient was achieved, thus suggesting its potential use as a preliminary screening tool to guide decision making in performing LGE-CMR in those cases in which its indication is uncertain.
